# Investigating the exposure of Iranian households to catastrophic health expenditure due to the need to purchase medicines

**DOI:** 10.1371/journal.pone.0214783

**Published:** 2019-04-26

**Authors:** Mohammadreza Amiresmaili, Zahra Emrani

**Affiliations:** 1 Medical Informatics Research Center, Institute for Futures Studies in Health, Kerman University of Medical Sciences, Kerman, Iran; 2 Department of Health Management and Economics, School of Public Health, Tehran University of Medical Sciences, Tehran, Iran; 3 Health Management Research Center, Institute for Futures Studies in Health, Kerman University of Medical Sciences, Kerman, Iran; University Complutense of Madrid, SPAIN

## Abstract

**Background:**

Catastrophic health expenditure (CHE) is an indicator used by the World Health Organization (WHO) to assess equity in households’ payments to the health system. In this paper, we prospectively calculated the population at risk of facing catastrophic expenditure due to purchasing three selected medicines (metformin, atorvastatin and amoxicillin) in Iran.

**Method:**

This study draws on the data set of the Iranian National Household Survey of 38244 households in Iran. CHE was calculated based on "capacity to pay" using different thresholds.

**Results:**

20, 16 and 3 households had to spend more than 40% of their capacity to pay on amoxicillin, atorvastatin and metformin respectively. Lowest priced generic (LPG) medicines were found more affordable than the original brand (OB) medicines. Age, literacy and gender of head of household, economic status, settlement, size and number of breadwinners in the households share important association with CHE.

**Conclusion:**

Requirement of these specific medicines for long-term may subject the Iranian households to CHE. The study demonstrates important and specific insights for health policy makers in Iran to protect the households from healthcare catastrophes.

## Introduction

Financial protection against health costs is one of the objectives of health systems determined by the World Health Organization (WHO) [1]. Therefore, protecting individuals against the financial outcomes of health care–related to the risk and uncertainty about the future health states- should be a consideration of any health system. Financial protection is evaluated through out-of-pocket payments, which are analyzed using two approaches: (a) catastrophic health expenditure (CHE) and (b) impoverishment [2,3]. Medical spending is “catastrophic” if it exceeds a certain proportion of “total expenditure” or "capacity to pay” [4,5], which forces households to decrease spending on basic needs [6]. Spending is “impoverishing” if it is so large that it pushes households under the poverty line [7]. Previous studies which have measured CHE. Yazdi-Feyzabadi et al. studied trends of impoverishing effects of out-of-pocket health expenditures [8] and CHE [9] in 2008–2014, retrospectively in Iran provinces. They used data set of the Iranian National Household Survey. Nekoei-Moghadam et al. also quantified CHE rate in Iran for 2008 by foregoing data set [10]. Kavousi et al. measured the households’ exposure to CHE, using the WHO questionnaire in zone 17 of Tehran province [11]. Some studies calculated CHE for a limited population of Qazvin [12] and Torbat-Heydarieh [13]. They used questionnaire to gather information.

A study in Poland considered the inequality and financial burden of medicine expenditures. The impoverishing effect of medicine payments applying two poverty lines (based on absolute and relative poverty), the incidence and intensity of catastrophic medicine expenditures were investigated. The OOP medicine expenditures was significantly unaffordable among the retired and chronically ill [14].

Regarding the methods of measuring affordability Niëns and Brouwer discussed. They noted the positive and negative aspects of retrospective, prospective and LPGW methods for affordability. They identified prospective method to calculate the number of households at risk of CHE. They have calculated medicine affordability as an example for each method [15]. Additionally in other studies Niëns et al. applied prospective method to quantify catastrophic expenditure [5] and impoverishment [16] due to spending on medicine.

Taking medicine is one of the main methods used to treat diseases [17], and thus affects households’ exposure to CHE [5]. According to the WHO, medicines account for 20–60% of health expenditure in developing and transitional countries. Up to 90% of people in developing countries pay for medicines by out-of-pocket payments, and this ranks second in family expenditures after food [18]. Studies have shown that medicine expenditures in Iran is rising more rapidly than other health care spending [19]. Health transitions in Iran in recent years have produced a financial burden on the health system, with the incidence of chronic diseases rising [20]. Patients with chronic health conditions usually have to pay for medicine for their entire life. Frequent medical examinations and medicines are necessary for their health. Sometimes, due to the household’s financial situation, patients may purchase medicines without referring to a physician [21]. According to Iran’s insurance system, a patient buying medicine with a doctor’s prescription should pay just 30% of the total cost, whereas without a prescription, the full cost must be paid out-of-pocket [22]. Additionally in case of purchasing the original brand (OB) medicines despite their higher price, insurance organization pays just 70 percent of the LPG equivalent price [23]. Difficulties in paying for medication and related expenditures impose heavy costs on households. Because studies have not considered catastrophic expenditure on medicines in Iran until now, and considering the increase in chronic diseases in recent years, this study aims to be the first one to prospectively compute the population at risk of facing catastrophic expenditure due to the purchase of three selected medicines (metformin, atorvastatin and amoxicillin) in Iran.

## Method

### Data collection

This cross-sectional study draws on data sets of the Iranian National Household Survey, conducted by the Statistical Center of Iran, for the year 2013. The sample size for this analysis—after removing households without food expenditure data—is 38244 households, of which 18854 lived in urban and 19390 in rural areas.

For our analysis, we required four kinds of data: (a) household income or consumption expenditure, (b) price of medicines, (c) thresholds to measure the financial burden on the households, and (d) prevalence rates for diseases.

“Income” or “consumption expenditure” data is needed to calculate the “capacity to pay” of each household. We used “household consumption expenditure” data as the basis for CTP, since it is a better proxy of welfare and its’ measurement is easier than the “household income” [24].

Medicine prices were obtained from Iran’s Pharmacists Association [25]. Out-of-pocket payments for medicines are typically 30% of the medicine price (when covered by insurance) plus dispensing fee on each prescription. Three medicines were selected: (a) amoxicillin, (b) metformin and (c) atorvastatin. These were reported as the most used medicines in 2013, by the Food and Drug Administration [26]. The other reason for choosing metformin and atorvastatin was that they are the main medicines prescribed for diabetes and high blood cholesterol, which are highly prevalent in Iran (8.6% and 41.6% respectively among 20–79 year olds) [27,28].

The burden of chronic illness is progressively increasing in Iran. It is notable that in Iran, diabetes was 9th cause of death in 2007, which reached 6th cause in 2017 ranking. High blood cholesterol is a risk factor for the 1st and 2nd causes of death (heart diseases and stroke) and some other chronic diseases [29]. These diseases have a considerable share in global burden of diseases too [30].

Table 1 shows the health conditions for which these medicines are prescribed, the treatment periods and the number of units per treatment course.

**Table 1:** Description of selected medicines.

Medicine	Health problems for which it is prescribed/prevalence in Iran	Form/number of doses per treatment	Treatment period (day)	Out-of-pocket (per treatment duration)
Amoxicillin(LPG)	Sinusitis / 53% [31]	Capsule (500 mg)/ 21	7	0.7 US dollar
Metformin(LPG)	Type 2 diabetes / 8.5%	Tablet (500 mg)/ 90	Life long	0.9 US dollar
Glucophage(OB)				4.4 US dollar
Atorvastatin(LPG)	High blood cholesterol / 41.6%	Tablet (10 mg)/ 30	Life long	0.6 US dollar
Lipitor(OB)				11.6 US dollar

### Data analysis

“Food expenditure” is a part of total household expenditures which is spent on food. All the other household spending is called “non-food expenditures”. In the other perspective, the “subsistence expenditure” is the expenditure to meet basic needs (like shelter and food) to survive in a society. All the other household spending is called “non-subsistence expenditure”.

We need “capacity to pay” as the denominator for calculating the rate of catastrophic health expenditure. In cases where the subsistence expenditure is more than food expenditure, CTP equals non-food expenditure, otherwise CTP equals non-subsistence expenditure [24,32].

$${\text{CTP}}_{\text{i}}={\text{Exp}}_{\text{i}}{-\text{Food}}_{\text{i}}\;\text{if}\;{\text{S}}_{\text{i}}>{\text{Food}}_{\text{i}}$$

$${\text{CTP}}_{\text{i}}={\text{Exp}}_{\text{i}}-{\text{S}}_{\text{i}}\;\text{if}\;{\text{S}}_{\text{i}}<={\text{Food}}_{\text{i}}$$

It is notable that the out of pocket payment (numerator of the ratio) consists of two parts: a) the health expenditure of the household in 2013, b) the out of pocket payments on medicine.

Accordingly, 10 scenarios were proposed (S1 Appendix). First, in three scenarios we study households in which only one member is sick. The second three scenarios include households with two ill members, or in which one member suffers from two diseases simultaneously. In the seventh scenario, all three medicines are used in a household. In the last three scenarios, households have to purchase OB medicines. We build these scenarios consulting medical specialists (a cardiologist, an endocrinologist, an infectious diseases specialist), they believed the scenarios brought in the paper are possible.

We chose four different thresholds (10%, 20%, 30% and 40%) according to Wagstaff**’**s study [33]. The share of out-of-pocket payments exceeding preset thresholds determines the CHE. To achieve the incidence of catastrophic medicine payments it is necessary to Subtract the percentages of households who exposed to CHE because of paying for health in 2013 (the numerator is household health expenditure) from the CHE rate after pay for medicines. The obtained gap shows the percentage of CHE caused by purchasing medicines.

The last part of the study evaluates the impact of several factors on catastrophic expenditure. For this part we did a logistic regression test. Regression analysis was conducted in SPSS version 19, which was based on the scenario in which metformin and atorvastatin were purchased (threshold 40%). The dependent variable was a dichotomous variable (0 = when households did not face 40% threshold of CHE due to the purchase of metformin and atorvastatin, and 1 = when households faced 40% threshold of CHE, after purchasing these two medicines).

## Results

Table 2 demonstrates the household characteristics. Among the heads of the households 12% were female, 72.7% were literate. About 50.7% of the sample lived in rural areas. There were more than one member with income in about 27.6% of the households.

**Table 2:** Descriptive statistics of the household characteristics (n = 38244).

Variable		Number	percentage
Gender of head of the household	Male	33640	88.0
	Female	4604	12.0
Literacy of head of the household	Literate	27789	72.7
	Illiterate	10455	27.3
Size of the household	Less than 5	28727	75.1
	5 or more	9517	24.9
Expenditure quintiles	Q1	7667	20.0
	Q2	7649	20.0
	Q3	7649	20.0
	Q4	7649	20.0
	Q5	7630	20.0
Settlement	Urban	18854	49.3
	Rural	19390	50.7
Age of head of the household	Lower than 40	14119	36.9
	40<age<60	15468	40.4
	60<age<80	7451	19.5
	More than 80	1206	3.2
number of earning members in the household	1	27669	72.3
	More than 1	10571	27.6

Table 3 shows the households exposed to catastrophic costs after purchasing medicines under different scenarios. First, the percentage of households already facing catastrophic costs is shown—those households who face catastrophic expenditure after paying for health costs. Then, the differences (gaps) caused by the purchase of medicines, which are the main CHE figures, are estimated. The third part of the table shows the expected number of households affected, using the prevalence rates. For example, 16 households spend more than 40% of their ability to pay on atorvastatin. When the proposed thresholds are 30%, 20% and 10%, the results are 32, 80 and 159 respectively. It clearly demonstrates that at a lower CHE threshold, higher number of households are found to experience healthcare catastrophe.

**Table 3:** Catastrophic out-of-pocket payments on medicines for Iran.

Thresholds	40%	30%	20%	10%	40%	30%	20%	10%	40%	30%	20%	10%
Scenarios of medicine consumption	Share of capacity to pay exceeds thresholds (percentage)				Percentage of CHE caused by purchasing medicines (gap)*				Number of households exposed to CHE**			
Before any medicine consumption	3.6	6.9	13.3	27.3								
After purchasing amoxicillin	3.7	7.1	13.9	28.6	0.1	0.2	0.6	1.3	20	41	122	264
After purchasing atorvastatin	3.7	7.1	13.8	28.3	0.1	0.2	0.5	1	16	32	80	159
After purchasing metformin	3.7	7.2	14	28.8	0.1	0.3	0.7	1.5	3	10	23	49
After purchasing amoxicillin &atorvastatin	3.8	7.4	14.4	29.8	0.2	0.5	1.1	2.5	-	-	-	-
After purchasing amoxicillin & metformin	3.9	7.6	14.6	30.4	0.3	0.7	1.3	3.1	-	-	-	-
After purchasing atorvastatin & metformin	3.9	7.5	14.5	30.2	0.3	0.6	1.2	2.9	-	-	-	-
After purchasing amoxicillin & atorvastatin & metformin	4	7.8	15.3	31.9	0.4	0.9	2	4.6	-	-	-	-
After purchasing Glucophage	4.6	9.1	17.5	37	1	2.2	4.2	9.7	33	72	137	315
After purchasing Lipitor	8.4	15	27.5	55.1	4.8	8.1	14.2	27.8	764	1289	2259	4423
After purchasing Glucophage & Lipitor	11.4	19.4	34.2	64.9	7.8	12.5	20.9	37.6	-	-	-	-

*"Percentage of CHE caused by purchasing medicines" equals "Percentage of CHE exceeding thresholds" minus "Share of capacity to pay exceeding thresholds before purchasing medicines".

** The CHE percentage, the sample size and the prevalence were multiplied when calculating the number of households exposed to CHE. For scenarios with more than one medicine to be purchased, the prevalence of different diseases were multiplied to calculate the probability of occurrence. Therefore, a smaller number was obtained. In this case, the CHE percentages are only reported.

The percentage of the households exposed to CHE increases with the number of illnesses in a family. If we assume the burden of 40%, CHE rate is 0.1 percent when purchasing amoxicillin, 0.2 percent for amoxicillin and atorvastatin, 0.4 percent for amoxicillin, atorvastatin and metformin.

The results also show that the catastrophic effects of medicines vary, especially between LPG (lowest priced generic) and OB medicines. For example when 0.1, 0.1 and 0.3 percent of the households face CHE because of using atorvastatin, metformin and both of them, in separate scenarios, the percentages increase to 1, 4.8 and 7.8 respectively when the households use OB medicines.

Table 4 shows the association between exposure to CHE and some households’ characteristics. It is found that the literacy, age and gender of head of the household and the size of the household have significant influence on CHE. The logistic test shows that households whose head is illiterate are approximately 1.519 times more likely than those with a literate head to face CHE. The age of head of the household seems to have a direct relation to CHE exposure, since the mean CHE increases with age. The size of the household is inversely associated with CHE: households with five or more members are 0.662 times less likely to expose with CHE than households with fewer members.

**Table 4:** Regression results for determinants of CHE.

Variables		Logistic regression results			
			OR (Lowest- highest)		
		OR	90% CI	95% CI	99% CI
Gender of head of the household	Male	1			
	Female	1.294	(1.141–1.469)*	(1.113–1.505)**	(1.062–1.578)***
Literacy of head of the household	Literate	1			
	Illiterate	1.519	(1.345–1.716)*	(1.314–1.756)**	(1.256–1.838)***
Size of the household	Less than 5	1			
	5 or more	0.662	(0.586–0.748)*	(0.573–0.766)**	(0.547–0.802)***
Expenditure quintiles	Q1	1			
	Q2	0.972	(0.840–1.125)	(0.817–1.157)	(0.773–1.222)
	Q3	1.029	(0.881–1.201)	(0.855–1.237)	(0.807–1.311)
	Q4	1.409	(1.202–1.635)*	(1.168–1.684)**	(1.102–1.784)***
	Q5	2.384	(2.052–2.769)*	(1.993–2.850)**	(1.885–3.015)***
Settlement	Urban	1			
	Rural	1.816	(1.646–2.002)*	(1.616–2.040)**	(1.558–2.116)***
Age of head of the household	Lower than 40	1			
	40<age<60	1.054	(0.931–1.192)	(0.910–1.221)	(0.869–1.279)
	60<age<80	1.951	(1.696–2.244)*	(1.651–2.305)**	(1.567–2.429)***
	More than 80	3.140	(2.570–3.838)*	(2.471–3.989)**	(2.292–4.301)***
number of earning members in the household	1	1			
	More than 1	0.852	(0.766–0.947)*	(0.750–0.967)**	(0.721–1.006)***

*<0.05,

**<0.01,

***<0.001

Rural households face with CHE 1.816 times more than urban households. The economic status of the households is an influential factor on CHE. The higher quintiles are more likely to face CHE than the lower quintiles. Additionally the test shows that the availability of more than one breadwinner in a household, decreases the risk of CHE 0.852 times.

## Discussion

A "Health Evolution Plan" was initiated in 2014 to overcome some of the main challenges of Iran’s health system [34]. Decreasing the number of households at risk of CHE and reducing out-of-pocket payments are from the main aims defined for health evolution plan. To reach the goals eight packages were developed. For example urban citizens under basic health insurance coverage must pay 6 percent of total hospital bill. This percentage reduces to 3 percent if rural population and urban residents living in cities with less than 20000 population, refer to public hospitals through the referral system. People with no basic health insurance were covered free of charge. Additionally financial protection of poor patients and incurable patients was considered in another package [35]. This plan also encompassed issues related to pharmaceutical costs. For example hospitals are mandated to provide medicines for inpatients. Just sometimes patients’ companions are asked to purchase medicines from hospital’s drugstore. They should not be asked to provide medicines from out of the hospital. However, the main focus of the plan was to lower out-of-pocket payments for inpatients, mainly those in hospitals belonging to the Ministry of Health and Medical Education [36,37]. Nevertheless, pharmaceutical costs still impose a heavy burden on households. Unfortunately, many people procure their required medicines from pharmacies, without obtaining a physician’s prescription [17,38,39]. Also, in Iran, people tend to use OB medicines, especially for serious illnesses, when recovery is slow or when their ability to pay is high. This study illustrated that, not only is medicine unaffordable for many households, but also the purchase of OB medicines increases the probability of exposure to catastrophic health expenditure several times compared to the purchase of LPG products. This finding has also been supported by Niëns et al. who calculated the impoverishment percentage in the Philippines after purchasing OB medicines to be 22%, compared to 7% for LPG equivalents [16].

Pharmaceutical expenditure was identified as an influential factor when it comes to catastrophic costs, by Nekoei et al. They reported the catastrophic costs of Iranian households to be 2.8%, by retrospectively applying the health spending method in 2008 [19]. The CHE surveys within provinces such as Yazd, Shiraz, Kerman, Tehran and others show an increase in inequality in CHE rates between provinces. The percentage of CHE in Iran for 2008–2014 increased [40].

Studies have also indicated that households who were subject to CHE share some features [41]. Accordingly, the present study showed that households with an illiterate head faced CHE 1.519 times more than households with a literate head. This might be explained by the fact that literacy is associated with higher socio-economic classes, they usually have two breadwinners (both couples work). Literacy is considered one of the social determinants of health, its effect on health status have been confirmed elsewhere [42].

This study revealed that when the household size is five or more, catastrophic expenditure is less probable. The results of some other studies carried out in this area also support this finding. Nekoei et al. showed that households with more than six members were less prone to catastrophic health expenditure [19]. Saber-Mahani et al. indicated a negative relationship between household size and catastrophic expenditure [43]. Having a large family in China is a protective factor against health costs, as identified by Li et al. [44]. This can be explained by the fact that in Iran, older households tend to have more members and more breadwinners than younger households. Meanwhile, there are usually small children in younger households, making them more prone to diseases and potentially less resistant in terms of economic status. This study showed the relationship between the gender of the head of household and CHE probability. The households with female head were at risk of catastrophic expenditure, as it was identified in previous study [45]. Despite the high number of women having jobs in Iran, more focus on this issue is still required. The other factor found relevant is age. As the age of the household’s head increases, the likelihood of exposure to CHE increases [46]. This is because of the reality that, with greater age, the risk of suffering from various diseases increases. This effect is exacerbated by the reduction in income at higher ages (due to retirement or disability). As indicated by Yardim et al., households with disabled or senior members are at greater risk of CHE [4].

The households’ income was another key factor. The likelihood of high quintiles to be exposed to CHE was higher than low quintiles, in this study. A study by Fazaeli demonstrated that high income households pay for health several times more than the low income ones. This increases risk of CHE, however they do not often end with health impoverishment due to their high income [47].

The high income households’ utilization of the expensive private sector services might be the reason. For example, a study on the catastrophic dental health expenditure (CDHE) reported that the rate of CDHE was greater in high income households [6]. However some literatures reported a negative relation between income levels and the CHE rates, which is because of the vulnerability of the poor against financial risks [10,41,48]. Löfgren indicated no significant relationship between households’ income and CHE [45].

The rural residence were significantly at risk of CHE. Different studies confirmed this result [4,41,44]. It might be resulted from their low income, low education levels or delay in diagnosis of their diseases [41].

## Conclusion

This study shows that taking medicine can make households subject to CHE (despite lower price of medicine compared with many other countries because of subsidy allocation). The age, literacy, and gender of the head of household and the size of the household are some factors that are found to be associated with CHE. The study demonstrates important and specific insights for health policymakers in Iran to protect the households from healthcare catastrophes, when long term requirement of these specific medicines raise potential risks for the Iranian households to experience CHE.

To reach Iran’s plan to decrease the catastrophic health costs to one percent, any part of the total health expenditure plays its role and has its own share. The results can be used to compare the catastrophic cost of medicines with the catastrophic cost of other treatments or health requirements. It may be helpful to make proper policies to reduce the cost of the section, which makes more catastrophic costs.

## Supporting information

S1 Appendix (DOCX)
