# Supplementary material for: Investigating the exposure of Iranian households to catastrophic health expenditure due to the need to purchase medicines
Source: PLoS One. 2019 Apr 26;14(4):e0214783. doi: 10.1371/journal.pone.0214783 (PMC6485892; doi:10.1371/journal.pone.0214783)
Supplement: S1 Appendix — (DOCX) [file pone.0214783.s001.docx]

Appendix 1. Scenarios included in the study

| Scenario number | Description | Medicines to be purchased |
| --- | --- | --- |
| 1 | Households are considered with one ill member | Metformin |
| 2 | Households are considered with one ill member | Atorvastatin |
| 3 | Households are considered with one ill member | Amoxicillin |
| 4 | This scenario includes households with two ill members, or in which one member suffers from two diseases simultaneously | Atorvastatin & Metformin |
| 5 | This scenario includes households with two ill members, or in which one member suffers from two diseases simultaneously | Amoxicillin &Atorvastatin |
| 6 | This scenario includes households with two ill members, or in which one member suffers from two diseases simultaneously | Amoxicillin & Metformin |
| 7 | In this scenario all three medicines are required in a household | Atorvastatin, Amoxicillin & Metformin |
| 8 | Households decide to buy original brand medicines | Glucophage™ |
| 9 | Households decide to buy original brand medicines | Lipitor™ |
| 10 | Households decide to buy original brand medicines | Glucophage™ & Lipitor ™ |
